# Large-Scale Indoor Camera Positioning Using Fiducial Markers

**DOI:** 10.3390/s24134303

**Published:** 2024-07-02

**Authors:** Pablo García-Ruiz, Francisco J. Romero-Ramirez, Rafael Muñoz-Salinas, Manuel J. Marín-Jiménez, Rafael Medina-Carnicer

**Affiliations:** 1 Departamento de Informática y Análisis Numérico, Edificio Einstein, Campus de Rabanales, Universidad de Coŕdoba, 14071 Córdoba, Spain; pgruiz@uco.es (P.G.-R.); rmedina@uco.es (R.M.-C.); 2 Departamento de Teoría de la Señal y Comunicaciones y Sistemas Telemáticos y Computación, Campus de Fuenlabrada, Universidad Rey Juan Carlos, 28942 Fuenlabrada, Spain; francisco.romero@urjc.es; 3 Instituto Maimónides de Investigación en Biomedicina (IMIBIC), Avenida Menéndez Pidal s/n, 14004 Córdoba, Spain

**Keywords:** indoor camera positioning, camera pose estimation, fiducial marker, video surveillance

## Abstract

Estimating the pose of a large set of fixed indoor cameras is a requirement for certain applications in augmented reality, autonomous navigation, video surveillance, and logistics. However, accurately mapping the positions of these cameras remains an unsolved problem. While providing partial solutions, existing alternatives are limited by their dependence on distinct environmental features, the requirement for large overlapping camera views, and specific conditions. This paper introduces a novel approach to estimating the pose of a large set of cameras using a small subset of fiducial markers printed on regular pieces of paper. By placing the markers in areas visible to multiple cameras, we can obtain an initial estimation of the pair-wise spatial relationship between them. The markers can be moved throughout the environment to obtain the relationship between all cameras, thus creating a graph connecting all cameras. In the final step, our method performs a full optimization, minimizing the reprojection errors of the observed markers and enforcing physical constraints, such as camera and marker coplanarity and control points. We validated our approach using novel artificial and real datasets with varying levels of complexity. Our experiments demonstrated superior performance over existing state-of-the-art techniques and increased effectiveness in real-world applications. Accompanying this paper, we provide the research community with access to our code, tutorials, and an application framework to support the deployment of our methodology.

## 1. Introduction

Estimating the positions of a large set of indoor fixed cameras is an open problem in computer vision. Installing a large set of cameras on the ceiling of a building (e.g., every two meters) would allow for a new set of applications in areas such as autonomous navigation, motion capture, augmented reality, video surveillance, and logistics [1,2,3,4,5].

This task is particularly challenging because of variable lighting, diverse architectural layouts, and the need for non-intrusive installations [6,7,8,9,10].

Existing solutions for fixed camera pose estimation are available for motion capture (MoCap) systems [11,12,13,14], where typically a small subset of cameras is placed around the tracking area where the movements of a person or object are tracked using reflective markers. The calibration of such methods requires a relatively large overlap between cameras, and they are typically designed to work at room level, not scaling well to large scenarios.

Camera pose estimation has also been tackled using various methodologies that are prominently divided into feature-based and marker-based techniques. Feature-based approaches are prevalent, including simultaneous localization and mapping (SLAM) [15] and structure from motion (SfM) [16]. SLAM often requires dense environmental features and substantial overlap between successive camera views, making it unsuitable for large indoor camera positioning. Although SfM is more focused on reconstructing the observed scene, the process requires estimating the camera poses. SfM techniques have expanded the camera pose estimation scope but continue encountering significant challenges. They rely heavily on distinct environmental features and require overlapping camera views for effective functionality. Despite offering a more suitable solution than SLAM methodologies for some scenarios, these constraints often limit the applicability of SfM in environments where such conditions are not readily met.

Recently, marker-based systems [17,18,19] have emerged as an alternative to feature-based approaches. These systems utilize artificial markers as reference points within the scene to facilitate marker and camera pose estimation, thereby simplifying the process and enhancing robustness by reducing the dependency on environmental features. However, it presents drawbacks, such as a reliance on unique marker identification, which makes them unsuitable for camera pose estimation in large indoor environments.

**Figure 1:**
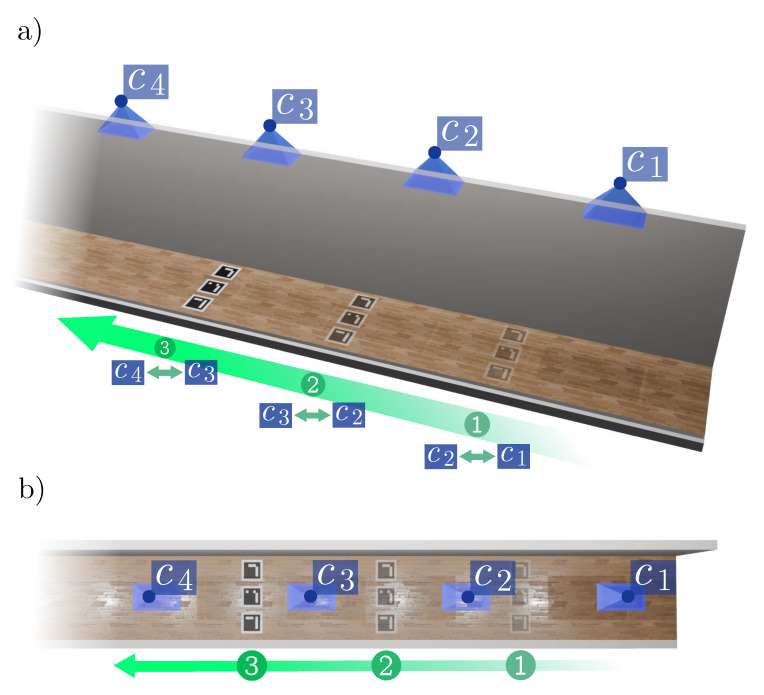
Overview of the camera pose estimation process using fiducial markers indoors. (**a**) Perspective view of a corridor showing four cameras (represented as blue pyramids) with the movement path of the fiducial markers to sequentially capture snapshots. (**b**) Top-down view of the corridor illustrating the placement and movement of markers relative to the camera positions.

This paper proposes a novel approach to estimate the pose of an arbitrarily large number of fixed cameras in indoor environments using fiducial markers. Our approach requires cameras to be visually connected by at least a small field of view so there is a path connecting all cameras throughout the environment. The relative pair-wise camera poses are estimated by placing a small subset of markers in the overlapping areas between them. This process is illustrated in Figure 1.

Once a picture of the markers is taken with a subset of connected cameras, the markers can be repeatedly moved to the next subset of connected cameras until all camera relationships are obtained. Consequently, our method requires only a very small subset of markers to be printed to accurately obtain the poses of any number of cameras. Then, a full optimization is run to minimize the reprojection error and other error terms, including spatial knowledge about the particular environment being mapped.

This is the first work, to our knowledge, that tackles the problem of large indoor fixed camera pose estimation using fiducial markers. Consequently, we had to create datasets to validate our proposal and compare it with state-of-the-art related works. Our experiments show the validity of the proposed method and demonstrate that most related works cannot solve the problem because the very limited field of view between the cameras makes them fail.

The rest of this work is organized as follows. Section 2 reviews related works, emphasizing significant methodologies and advancements in camera pose estimation. Section 3 details the methodology of our proposed approach, elaborating on the technical innovations. Section 4 describes the experimental setup and discusses the results obtained, which validated the effectiveness of our approach. Section 5 concludes this paper by discussing the implications of our findings and suggesting avenues for future research.

## 2. Related Works

Camera pose estimation encompasses various methodologies designed to determine the orientation and location of cameras within a specific environment. Initially similar to object positioning, which offers diverse approaches, as detailed in studies like the ones discussed by Guan et al. [20] on 6DoF object pose estimation using cutting-edge technologies, the practical requirements differ notably. In real-world scenarios, particularly indoors, cameras are often placed in less accessible areas, such as ceilings, complicating direct object positioning methods. Therefore, methodologies that perform well in controlled or laboratory settings, such as those employed in MoCap systems [11,12,13,14], may not be directly applicable to more general indoor camera positioning.

One of the techniques used regardless of camera location to estimate the camera pose is based on keypoint information. This approach involves detecting and matching distinctive image features (keypoints) to estimate relative camera poses. Known as photogrammetry, this technique enables the extraction of dense 3D geometric information from overlapping stereoscopic images, facilitating the accurate reconstruction of real-world cameras [21]. There are two primary approaches in multi-camera pose reconstruction via keypoint-based information.

The first approach is simultaneous localization and mapping (SLAM), which is tailored for cameras that navigate an environment. Recent developments, such as those proposed by Romero-Ramirez et al. in [22] and by Campos et al. [23], demonstrate the efficacy of this methodology for moving cameras in dynamic environments. However, SLAM solutions excel particularly well when numerous frames, which can be treated as different camera views, are well-correlated, orderly, and have sufficient overlap to reconstruct the scene accurately. This level of correlation and overlap is not always achievable in real-world indoor camera setups, where cameras are typically arranged efficiently but not necessarily optimally for SLAM requirements.

The second approach to multi-camera pose reconstruction utilizes structure from motion (SfM) techniques, which comprise a significant branch of multi-camera photogrammetry. As outlined by Ullman [24], SfM is a robust methodology for reconstructing 3D structures from 2D image sequences by simultaneously estimating camera parameters and scene structure. Traditional SfM pipelines include keypoint detection and matching, bundle adjustment, and loop closure mechanisms, which are essential for precise reconstructions. Several software solutions exist, such as Pix4D [25], OpenDroneMap [26], and COLMAP [27]. Nevertheless, these methods heavily rely on the environment having distinguishable features and adequate overlap between camera views to function effectively.

Recently, deep learning has enhanced SfM by improving the feature detection and matching, and directly predicting the depth and pose. Convolutional neural networks (CNNs) and recurrent neural networks (RNNs) have been employed to improve the robustness and accuracy of SfM pipelines. Techniques such as those proposed by Wang et al. [28] and Ren et al. [29] leverage deep learning models to refine the reconstruction process, leading to better performance in challenging scenarios. Nonetheless, these approaches still depend on the distinguishable features of the environment and their presence in overlapping camera views.

A novel alternative that leverages deep learning to enhance traditional SfM methods is Gaussian splatting. This technique, originally proposed by Kerbl et al. [30], utilizes neural networks to model scenes with a continuous volumetric function. Gaussian splatting can handle complex scenes with remarkable accuracy and provides a smooth reconstruction process, thereby showing significant advancements over traditional discrete representations. Tools such as the one provided by PolyCam [31] facilitate rapid testing and deployment of this method. Nevertheless, the efficacy of Gaussian splatting is still dependent on the quality of the underlying SfM reconstruction from which it derives.

Alternatively, some researchers proposed using fiducial markers (such as ArUco [32]) to improve the pose estimation process. Marker-based systems are particularly beneficial in controlled environments where markers can be strategically placed to ensure visibility and minimize ambiguity. These systems circumvent the need for abundant environmental features and extensive overlapping between camera views. They require only the presence of at least one common marker within the scope of each camera pair to accurately position every camera in a shared reference system. In [18], the authors propose a novel SLAM method that gracefully combines keypoints and fiducial markers, which represent long-term features unaffected by lighting conditions.

Another such method is MarkerMapper [17], which can be seen as an SfM method that uses fiducial markers instead of keypoints. MarkerMapper aims to estimate the position of a moving camera in an indoor environment. To do so, a large set of fiducial markers is placed on the walls and ceiling, and their position is estimated from pictures taken by a moving camera. It allows for the creation of a map of markers so that later, the position of another camera can be estimated by an image showing a single marker. This method is mainly conceived as a cheap localization method in robotic and virtual reality applications with low resources.

Although MarkerMapper focuses on estimating marker poses, not camera poses, they are computed as part of the optimization process. Thus, we can use this method to compare with our proposal. However, this method has several drawbacks. First, a large set of unique markers is needed to perform the reconstruction, which is especially challenging as the environment to map becomes larger. Second, the method relies solely on marker reprojection errors, omitting potential enhancements from additional information, such as control points or assumptions regarding the actual locations of cameras and markers, which may be easy to know in advance and beneficial for the reconstruction.

This paper proposes a novel approach to estimating the poses of cameras in large indoor environments from images showing fiducial markers that, unlike MarkerMapper, can be reused. Our method can obtain the poses of an unlimited number of cameras using as low as nine different markers, as shown in our experiments. In addition, our method allows for including knowledge about the environment to improve the reconstruction results, such as control points and certain restrictions about the camera and marker distribution.

## 3. Proposed Method

This section outlines the proposed methodology for estimating the positions of indoor cameras using fiducial markers. Our method estimates the relative positions between pairs of cameras by analyzing the markers simultaneously seen in the overlapping field of view. Given a group of cameras sharing a common field of view, a set of markers is placed, and images are captured with the cameras. For each camera, the relative positions of the visible markers relative to the camera are computed and used to obtain the pair-wise relationships between all the cameras involved. As a consequence, it creates a local pose graph connecting the cameras. The local pose graph errors are optimized by jointly minimizing the projection errors of the markers.

The process is repeated for all camera groups, ensuring at least one common camera between groups. This approach lets us connect groups with at least one camera, creating a global graph of poses so that there is a connection between any pair of cameras in the graph. In the global pose graph, the minimum expanding tree with the lowest pose error is selected and the camera poses are used as the starting solution for global optimization. The optimization aims to reduce the projection errors of the markers observed in the images and restrictions to mitigate the *doming effect* [33] and ensure proper scaling.

### 3.1. Mathematical Definitions

This section provides some initial concepts and mathematical definitions used in this paper.

First, let us denote by $p_{a}\in R^{3}$ a three-dimensional point in an arbitrary reference system *a*. To express such a point in another reference system, i.e., $p_{b}$, it must undergo a rotation followed by a translation. Let us denote by

(1) $$\left(r,t\right)\;|\;r,t\in R^{3},$$

the rotation and translation $r=(r_{x},r_{y},r_{z})$ and $t=(t_{x},t_{y},t_{z})$. The rotation matrix R can be obtained from r using the Rodrigues’ formula [34]:

(2) $$R=I_{3\times 3}+\bar{r}\sin\alpha +{\bar{r}}^{2}\left(1-\cos\alpha \right),$$

where $I_{3\times 3}$ is the identity matrix and $\bar{r}$ denotes the matrix

(3) $$\bar{r}=\left[\begin{array}{ccc} 0 & -r_{x}/\alpha & r_{y}/\alpha \\ r_{z}/\alpha & 0 & -r_{x}/\alpha \\ -r_{y}/\alpha & r_{x}/\alpha & 0 \end{array}\right]$$

such that $\alpha ={\left||r|\right|}_{2}$.

Then, using t, we can obtain the 4×4 SE(3) homogeneous transformation matrix

(4) $$\theta_{ba}=\left[\begin{array}{cc} R & t^{⊤}\\ 0 & 1 \end{array}\right]$$

that transforms points from *a* to *b* as follows:

(5) $$\left[\begin{array}{c} p_{b}^{⊤}\\ 1 \end{array}\right]=\theta_{ba}\left[\begin{array}{c} p_{a}^{⊤}\\ 1 \end{array}\right]$$

Consequently, the transformation from *c* to *b* ($\theta_{bc}$) followed by the transformation from *b* to *c* ($\theta_{cb}$) can be concatenated as a matrix multiplication into a unique transformation $\theta_{ac}$ as follows:

(6) $$\theta_{ac}=\theta_{ab}\theta_{bc}.$$

A three-dimensional point $p\in R^{3}$ will project at a camera’s pixel $u\in R^{2}$ using the pinhole camera model. Assuming that the camera parameters are known, the projection can be obtained as a function Ψ:

(7) u=Ψ(δ,θ,p),

where δ refers to the camera’s intrinsic parameters and θ refers to the camera pose from which the image was acquired, i.e., the transformation that moves a point from an arbitrary reference system to the camera one.

### 3.2. Cameras, Markers, and Groups

We shall define a camera $c_{j}$ and the set of cameras *C* by

(8) $$C=\{c_{j}|j=1\ldots n_{c}\}.$$

Our ultimate goal is to estimate the pose $\theta_{c_{j}}$ of each camera, i.e., a transformation that moves points from the global reference system (grs) to the camera reference system (crs).

To solve our problem, we create camera groups:

(9) $$G=\{g_{i}|i=1\ldots n_{g},g_{i}\cap g_{j}\neq \emptyset ,\forall i,j\}$$

**Figure 2:**
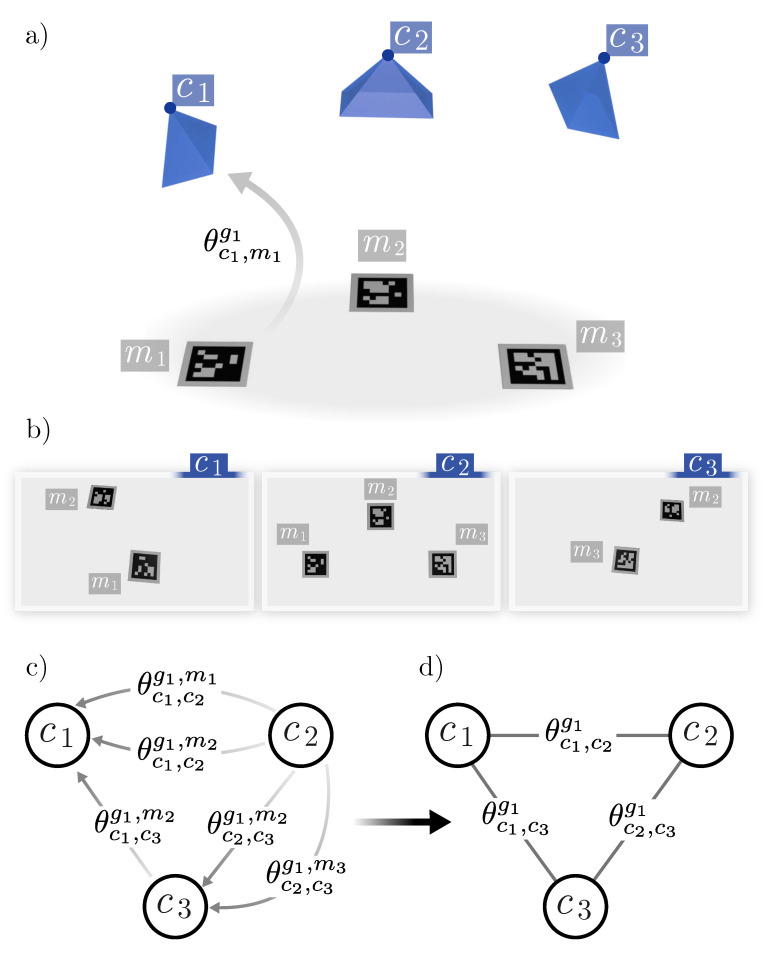
Camera groups. (**a**) Example of a camera group, where the cameras are represented as blue pyramids. (**b**) Images captured with these cameras. (**c**) Pose quiver for this example. (**d**) Camera poses graph for this example.

A group $g_{i}=\left\{c_{j}\in C\right\}$ consists of a set of cameras with overlapping views. We place markers in that area and capture an image with each one of the cameras of the group (Figure 2a). We shall denote

(10) $$M\left(g_{i}\right)=\left\{m_{l}|l=1\ldots n_{g_{i}}\right\}.$$

as the set of makers placed in the group $g_{i}$, where $m_{l}$ is a marker.

The projections of the four corners of a marker allow us to estimate the transform $\theta_{c_{j},m_{l}}^{g_{i}}$ from the marker reference system (mrs) to the reference system of the camera $c_{j}$ by minimizing the reprojection error from 3D to 2D point correspondences [35,36]. Let us define the following:

(11) $$\begin{array}{cccc} m_{l,1}=( & s/2, & -s/2, & 0\;),\\ m_{l,2}=( & \begin{array}{c} s/2, \end{array} & s/2, & 0\;),\\ m_{l,3}=( & -s/2, & s/2, & 0\;),\\ m_{l,4}=( & -s/2, & -s/2, & 0\;), \end{array}$$

as the corners of a square planar marker $m_{l}$, with sides of length *s*. These points are in the marker reference system, which is at its center. Also, we shall denote by

(12) $$m_{l,k}^{g_{i},c_{j}},k=1\ldots 4,$$

the projections of the corner markers $m_{l}$ in the camera $c_{j}$ of a group $g_{i}$.

When a marker is visible in two cameras of a group, it provides an estimation of the relative pose between them. Our method relies on that idea to create a graph where nodes represent cameras and edges represent their relative poses. For each group, a subgraph is created, and the set of all groups creates a global graph that describes the pair-wise relationship of all the cameras in the environment. The rest of this section explains in detail how the process is carried out.

### 3.3. Group Optimization

As already indicated, we call *group* $g_{i}$ a set of cameras with a common field of view. A set of markers $m_{l}$ is placed in the common field of view and captures images from all the cameras. Figure 2 helps to understand the explanation of this section. In the example, the first group ($g_{1}$) has three cameras $c_{1}$, $c_{2}$, and $c_{3}$ and three markers $m_{1}$, $m_{2}$, and $m_{3}$. As shown in the picture, not all cameras need to see all markers, but they must have at least a common marker.

We shall denote by

(13) $$O\left(g_{i}\right)=\left\{\left(c_{j},m_{l}\right),c_{j}\in g_{i},m_{l}\in M\left(g_{i}\right)\right\}$$

the set of camera–marker observations. In our example (Figure 2b),

(14) $$O\left(g_{1}\right)=\left\{\left(c_{1},m_{1}\right),\left(c_{1},m_{2}\right),\left(c_{2},m_{1}\right),\left(c_{2},m_{2}\right),\left(c_{2},m_{3}\right),\left(c_{3},m_{2}\right),\left(c_{3},m_{3}\right)\right\}$$

For every marker $m_{l}$ detected by a camera $c_{j}$, it is possible to obtain the transformation $\theta_{c_{j},m_{l}}^{g_{i}}$ of moving points from the marker to the camera using the 3D–2D correspondences of its four corners [35,36]. Then, using the common markers in the camera pairs, it is possible to obtain their relative pose as follows:

(15) $$\theta_{c_{j},c_{r}}^{g_{i},m_{l}}=\theta_{c_{j},m_{l}}^{g_{i}}{\left(\theta_{c_{r},m_{l}}^{g_{i}}\right)}^{-1},$$

so that $\theta_{c_{j},c_{r}}^{g_{i},m_{l}}$ represents the transformation from camera $c_{j}$ to $c_{r}$, and ()^−1^ denotes the inverse transformation.

As represented in Figure 2c, using all possible combinations, a pose quiver is obtained, where nodes represent camera poses and edges represent pair-wise camera relationships. From all possible edges between cameras, the one with the lowest reprojection error is selected, obtaining a pair-wise pose graph $\theta_{c_{j},c_{r}}^{g_{i}}$ between the cameras of the group (Figure 2d).

The next step is to refine the poses by minimizing the reprojection errors of all markers across all the cameras in the group. To do so, all cameras and markers must refer to a common reference system. Thus, we assume that one of the cameras (e.g., $c_{1}$) is the common reference system. Then, the transformation of all cameras $\theta_{c_{j}}^{g_{i}}$ can be easily obtained as follows:

(16) $$\theta_{c_{j}}^{g_{i}}=\left\{\begin{array}{ccc} 0 & if & j=1\\ \theta_{c_{1},c_{j}}^{g_{i}} & \forall i\neq 1 & \end{array}\right.$$

Please note that $\theta_{c_{j}}^{g_{i}}=0$ means that the resulting 4×4 matrix is the identity matrix (see Section 3.1).

Similarly, the transformation of each marker with respect to the common reference system $\theta_{m_{l}}^{g_{i}}$ is obtained as follows:

(17) $$\theta_{m_{l}}^{g_{i}}=\theta_{c_{j}}^{g_{i}}\theta_{c_{j},m_{l}}^{g_{i}}.$$

Therefore, the reprojection error of the markers visible in the camera group $g_{i}$ can be computed as the difference between the observed marker corner $m_{l,k}^{g_{i},c_{j}}$ and its estimated projection:

(18) $$\begin{array}{cc} E_{rp}\left(g_{i}\right) & =\sum_{\left(c_{j},m_{l}\right)\in O\left(g_{i}\right)}\\ \; & \;\sum_{k=1}^{4}{∥m_{l,k}^{g_{i},c_{j}}-\Psi \left(\delta_{c_{j}},{\left(\theta_{c_{j}}^{g_{i}}\right)}^{-1}\theta_{m_{l}}^{g_{i}},m_{l,k}\right)∥}^{2}. \end{array}$$

Therefore, in the example of Figure 2, the camera poses of the graph can be refined by minimizing the reprojection error

(19) $$\begin{array}{cc} \; & ({\dot{\theta }}_{c_{1}}^{g_{1}},{\dot{\theta }}_{c_{2}}^{g_{1}},{\dot{\theta }}_{c_{3}}^{g_{1}},{\dot{\theta }}_{m_{1}}^{g_{1}},{\dot{\theta }}_{m_{2}}^{g_{1}},{\dot{\theta }}_{m_{3}}^{g_{1}})=\\ \; & \;\underset{\theta_{c_{1}}^{g_{1}},\theta_{c_{2}}^{g_{1}},\theta_{c_{3}}^{g_{1}},\theta_{m_{1}}^{g_{1}},\theta_{m_{2}}^{g_{1}},\theta_{m_{3}}^{g_{1}}}{arg min}E_{rp}\left(g_{1}\right) \end{array}$$

using the sparse Levenberg–Marquardt algorithm.

Once the camera group $g_{i}$ is optimized, a refined local pose graph is created by obtaining the camera pair-wise relationships from the optimized results as follows:

(20) $${\dot{\theta }}_{c_{j},c_{r}}^{g_{i}}={\left({\dot{\theta }}_{c_{j}}^{g_{i}}\right)}^{-1}{\dot{\theta }}_{c_{r}}^{g_{i}}$$

### 3.4. Global Graph

After processing all groups individually, the local pose graphs obtained are merged into a global pose graph from which initial pose estimations for each camera can be obtained. To do so, first, one of the cameras is selected as the global reference system (e.g., $c_{1}$) and then the shortest paths to all other nodes are calculated. An initial global camera pose ${\hat{\theta }}_{c_{j}}$ is calculated from its path. For instance, if the shortest path from camera $c_{1}$ to camera $c_{10}$ is $\left(c_{1},c_{3},c_{8},c_{10}\right)$, then

(21) $${\hat{\theta }}_{c_{10}}=\theta_{c_{1},c_{3}}\theta_{c_{3},c_{8}}\theta_{c_{8},c_{10}}$$

where $\theta_{c_{j},c_{r}}$ are the pair-wise relations between cameras expressed in the graph’s edges.

Given the initial camera poses from Equation (21), the initial marker pose in the global reference system can be computed as follows:

(22) $${\hat{\theta }}_{m_{l}}^{g_{i}}=\theta_{c_{j}}\theta_{c_{j},m_{l}}^{g_{i}}$$

where $c_{j}$ is any of the cameras that observe the $m_{l}$ marker in group $g_{i}$. Please notice here that markers do not need to have unique IDs in our work since each marker ID is tied to a group. As a consequence, we can use the same marker in different groups.

The camera and marker poses previously calculated in Equations (21) and (22) are in an arbitrary reference system (camera $c_{1}$). However, in practical applications, it is desirable to refer all camera and marker poses to a CAD drawing (or map) of the building. For this purpose, control points are used. A control point $\left\{p_{c_{j}}\in R^{3}\right\}$ is the true position of camera $c_{j}$ in the map, and by Π, we denote the set of control points. Given at least three control points, the Horn [37] algorithm is used to find the best-fitting rigid transformation $\theta_{cp}$ by moving the camera and marker poses to the map reference system:

(23) $$\theta_{c_{j}}=\theta_{cp}{\hat{\theta }}_{c_{j}}$$

and

(24) $$\theta_{m_{l}}^{g_{i}}=\theta_{cp}{\hat{\theta }}_{m_{l}}^{g_{i}}$$

If no control points are indicated, $\theta_{cp}$ can be regarded as zero and the Equations (23) and (24) have no effect, i.e., $\theta_{c_{j}}={\hat{\theta }}_{c_{j}}$ and $\theta_{m_{l}}^{g_{i}}={\hat{\theta }}_{m_{l}}^{g_{i}}$

### 3.5. Global Optimization

The camera and marker poses calculated using Equations (23) and (24) are initial estimations obtained from the global graph and the control points. In this final step, these estimations are further refined, combining multiple objectives into a single global error $E_{g}$ by using appropriate weights:

(25) $$E_{g}=E_{rp}+E_{cc}+E_{cm}+E_{cp}$$

The error term $E_{rp}$ represents the reprojection error of all visible markers across all the groups and is calculated as follows:

(26) $$E_{rp}=w_{rp}\sum_{G}E_{rp}\left(g_{i}\right),$$

where $E_{rp}\left(g_{i}\right)$ is defined in Equation (18) and $w_{rp}$ is a normalization term used so that $E_{rp}=1$ when all errors $E_{rp}\left(g_{i}\right)$ are equal to one. It is calculated as the inverse of the total number of projected points, i.e.,

(27) $$w_{rp}=1/\left(\sum_{G}\sum_{\left(c_{j},m_{l}\right)\in O\left(g_{i}\right)}\sum_{k=1}^{4}1\right).$$

The normalization factor allows for combining the different error terms independently of the number of optimized markers, cameras, or control points.

The error term $E_{cc}$ is optional and is employed to enforce coplanarity in cameras that are indeed known to be in the same plane. This is typical in indoor scenarios, where cameras are placed on the ceiling. Let us use $\Theta_{o}=\left\{c_{j}\right\}$ to denote a set of cameras that are all in the same plane. If there are several different planes, which is a typical situation in indoor scenarios when modeling multiple floors, there is a $\Theta_{o}$ set for each plane. Then, $\Theta =\{\Theta_{o}$} is the set of all $\Theta_{o}$ sets and the error term is calculated as follows:

(28) $$E_{cc}=w_{cc}\sum_{\Theta_{o}\in \Theta }\sum_{\theta_{c_{j}}\in \Theta_{o}}{d_{t}}^{2}\left(t\left(\theta_{c_{j}}\right),P\left(\Theta_{o}\right)\right),$$

where $P(\Theta_{o})$ is the fitting plane determined from the camera poses in the set $\Theta_{o}$, and $d_{t}$ denotes the Euclidean distance between the translational component t (Equation (1)) of the camera pose $\theta_{c_{j}}$ and the plane. In this case, the normalization factor $w_{cc}$ is such that $E_{cc}=1$ when all the distances are equal to 1 centimeter. A pixel of error in $E_{rp}$ is assimilated to a centimeter of error in $E_{cc}$.

Similarly, the error term $E_{cm}$ is optional and is employed to enforce coplanarity in markers that are indeed known to be in the same plane. This is a typical situation in indoor scenarios where markers are placed on the floor. The term is calculated as follows:

(29) $$E_{cm}=w_{cm}\sum_{\Upsilon_{o}\in \Upsilon }\sum_{\theta_{m_{l}}^{g_{i}}\in \Upsilon_{o}}\sum_{k=1}^{4}{d_{t}}^{2}\left(t\left(\theta_{m_{l},k}^{g_{i}}\right),P\left(\Upsilon_{o}\right)\right)$$

where $P(\Upsilon_{o})$ is the fitting plane of coplanar markers $\Upsilon_{o}$, and $d_{t}$ denotes the Euclidean distance between the translational component of the marker pose $\theta_{m_{l},k}^{g_{i}}$ and the plane. $w_{cm}$ is such that $E_{cm}=1$ when all the distances are equal to 1 centimeter.

Finally, the optional term $E_{cp}$ refers to the use of control points, which forces cameras to be at the specified locations using the term

(30) $$E_{cp}=w_{cp}\sum_{p_{c_{j}}\in \Pi }∥p_{c_{j}}-t\left(\theta_{c_{j}}\right){∥}^{2}.$$

As can be observed, the errors consist of measuring the distance between the camera positions and their ground-truth positions. $w_{cp}$ is such that $E_{cp}=1$, i.e., all the distances equal 1 mm, and thus, more precision is required for these points than in the previous two cases.

## 4. Experiments

This section explains the experiments that were carried out to validate our proposal. To our knowledge, there are no public datasets to test our method, so we had to create them. We designed five experiments, with some using synthetically created datasets and the rest using real datasets created in our building.

The synthetic datasets were created using Blender [38] (version 4.0), which facilitated the generation of precise camera positions and markers that served as our ground truth. First, a virtual scene was created, including walls, floors, and lighting conditions, that mimicked a real-world environment. Then, simulated cameras were strategically placed, ensuring a realistic perspective. Afterward, each fiducial marker was meticulously positioned on top of the floor, presented as square planes, to emulate their typical usage in real-world settings. To compare the results of our method with MarkerMapper [17], we placed a set of unique synthetic ArUco markers. However, please notice that our method does not need a large set of unique markers to be employed, i.e., the mapping can be done using a small subset of markers that are reused.

**Figure 3:**
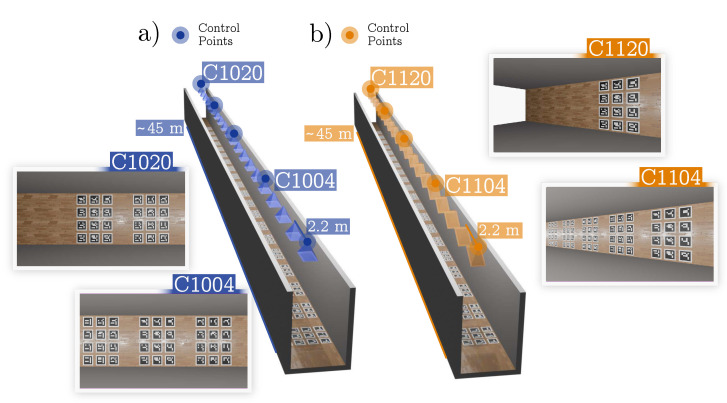
**Synthetic corridor datasets**. (**a**) Representation of the synthetic corridor (Dataset A), where cameras are shown as blue pyramids. The left images show two of the camera views generated with Blender. (**b**) Representation of Dataset B, along with some sample images.

**Figure 4:**
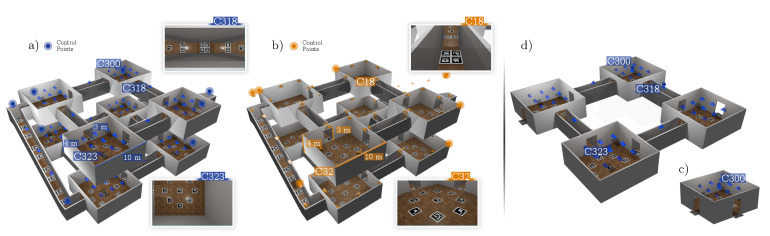
**Synthetic duplex datasets**. (**a**) Dataset A. (**b**) Dataset B. (**c**) First room of Dataset A. (**d**) First floor of Dataset A. See text for further details.

We first modeled a long corridor (Figure 3). It was a simple scenario in which we tested different configurations of cameras and markers. The results are shown in Section 4.1. Then, in Section 4.2, we show the modeling of a more complex scenario that represented an apartment of eight rooms distributed on two floors and connected by hallways (Figure 4).

**Figure 5:**
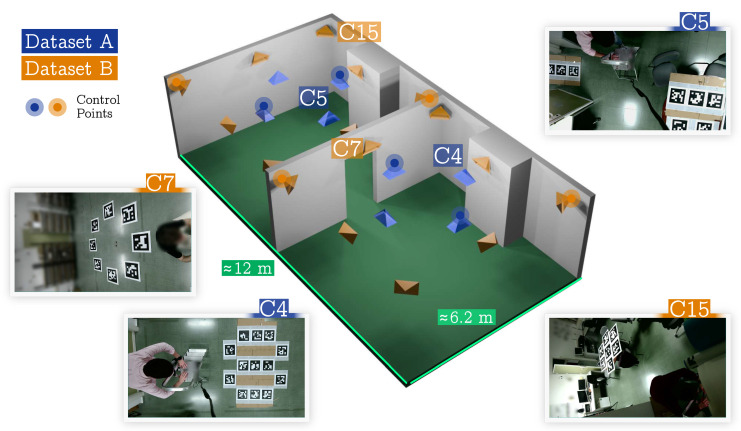
**Adjacent rooms datasets**. Three-dimensional reconstruction of the environment for visualization purposes. The camera configurations of both datasets are represented by blue (Dataset A) and orange (Dataset B) pyramids.

Additionally, two other experiments with real data from our lab were created. In this case, the ground-truth camera positions were manually obtained. These datasets were particularly interesting for evaluating the ability of our method to generalize correctly in real environments. In the first real dataset (Section 4.3), we reconstructed two adjacent laboratories connected by a room (Figure 5). The second real dataset (Section 4.5) consisted of the entire building floor of our lab, which comprised four large corridors connected by a large hall. For these datasets, only twelve ArUco markers were employed. They were printed on paper and moved along the building.

The experiments utilized a computer with an AMD Ryzen 7 5800U processor and Radeon graphics operating on an Ubuntu 20.04.5 OS. The average processing time per dataset was 50 min. We would also like to indicate that we used the ArUco detector [39], which was shown to be the most robust against various image artefacts, such as poor illumination and blurring [40]. Nevertheless, blurring was not a problem in this scenario because the cameras were fixed, and we could ensure good illumination during the recording session since it was a controlled environment.

The following methods were considered to compare the performance of our method with other approaches. First, MarkerMapper [17], which is a method used to solve the inverse problem: estimating the position of a set of markers from a set of images. However, as a subproduct of the process, MarkerMapper obtains the pose of the images. Its main drawback is that each marker must be unique, i.e., one cannot reuse markers. Since this is impractical in large real-world scenarios, MarkerMapper was only applied to the synthetic datasets (see Section 4.1 and Section 4.2), as well as for the adjacent rooms datasets (see Section 4.3). Second, in the structure from motion category, we tested Pix4D [25], OpenDroneMap [26], and COLMAP [27]. Finally, we extended our comparisons to include PolyCam [31], which is a novel reconstruction method based on 3D Gaussian splatting.

The rest of this section explains in detail each dataset and the results of our method in each one of them.

### 4.1. Synthetic Corridor

In this experiment, we modeled a long corridor that covered 90 m^2^, with cameras on the ceiling (see Figure 3). The cameras had minimal overlapping in their field of view, ensuring that each one overlapped with its nearest neighbors only. In total, there were 20 cameras placed at a 2.2 m distance from each other, with a resolution of 1920 × 1080 px, a focal length of 1173 px, and their FOV was ≈79°. The scenario was such that 12 markers of 32 cm were placed below every camera.

Using this scenario, we created two datasets, A and B, by changing the orientations of the cameras. In Dataset A (Figure 3a), the cameras were placed completely facing down, while in Dataset B (Figure 3b), they were slightly rotated to have a wider view of the corridor.

We examined the robustness of our method by conducting ablation experiments to evaluate the effects of the different optimization terms on the reconstruction quality. These experiments include the optimization of the $E_{rp}$ term only, along with all possible combinations of errors. The results are presented in Table 1, where column $E_{t}$ shows the translational error of the cameras, and column $E_{r}$ represents the rotational error. Our method achieved millimeter-level translation errors and very low angular errors, demonstrating its high precision. However, it is notable that Dataset B exhibited higher errors when only using the term $E_{rp}$. This could be attributed to the oblique arrangement of the cameras relative to the markers, which posed a more challenging reconstruction task.

Additionally, for Dataset A, including extra optimization terms did not significantly improve the reprojection error alone, resulting in reconstructions that were nearly of the same quality as the original. In contrast, for Dataset B, this inclusion resulted in a notable improvement in the translational errors. It is important to highlight that for both datasets, the rotational errors remained stable across all experimental configurations.

### 4.2. Synthetic Duplex

In this experiment, we designed a more challenging environment with multiple rooms and corridors, cycles, variability in camera arrangement, and two different floors (see Figure 4). The environment consisted of eight rooms and ten corridors that covered an area of 1077 m^2^ and was structured on two floors connected by ramps. Using the same structure, we created two datasets using different cameras and marker arrangements.

The first one, Dataset A, consisted of 192 makers of 80 cm and 90 cameras with a resolution of 1920 × 1080 px, a focal length of 1600 px, a FOV of ≈62°, and were placed facing down. The main change in Dataset B was that the rooms’ cameras were placed on the walls and pointed to the center. To properly cover the entire area with some overlap, we needed 94 cameras with a focal length of 480 px and a FOV of ≈127°.

To better understand the evolution of the errors as the complexity increased, each dataset was divided into three complexity levels: a single room, a single floor, and the whole scenario (the two connected floors). For Dataset A, these divisions are illustrated in Figure 4c, Figure 4d, and Figure 4a, respectively, facilitating a focused evaluation of the method’s performance across varying levels of structural complexity.

To test the performance of our method across these datasets, we performed a set of ablation experiments to test the effects of different optimization terms, as was done for the synthetic corridor datasets. The results are illustrated in Table 2.

For the first room, our method yielded submillimeter errors and very low angular errors. However, as the complexity of the dataset increased, the errors increased up to centimeter levels, demonstrating that the inherent complexity of the data directly impacted the accuracy of the computed solutions. Furthermore, it was observed that the solutions for Dataset B were less precise with respect to the translational errors. Nonetheless, our rotational errors remained below one degree in all cases. Specifically, for translational errors, we began at millimeter-level precision for the first room and increased to as much as several centimeters for the complete Dataset B.

Regarding the use of different optimization term configurations, we concluded that our method, when using all configured optimization terms jointly, achieved better reconstruction in every case compared with using only the reprojection errors, particularly in terms of the translational errors. The exceptions were for Dataset A, where we obtained similar reconstruction quality as when using only the reprojection errors, and for rotational errors, where we consistently achieved similarly low errors across all the tested configurations.

### 4.3. Adjacent Rooms

This was the first experiment with real data. In it, we aimed to find the position of the cameras placed in a real scenario comprised of two adjacent rooms (≈72 m^2^), as shown in Figure 5. For the experiment, we tested two different camera configurations (Dataset A and B), which are shown in the figure in blue and orange. For Dataset A (blue color), eight cameras were placed on the ceiling facing down, looking at a set of 25 markers of 21.7 cm placed on the floor. The cameras had a resolution of 1920 × 1080 pix, a focal length of ≈1108 pix, and a FOV of ≈84°. For Dataset B (orange color), a total of 16 cameras of the same type as in Dataset A were placed on the walls, looking at 16 markers with a marker size of 40 cm. Figure 5 also shows some of the images of the datasets.

While ground truth camera positions were manually obtained, it was impossible to know their orientation accurately. Consequently, we only evaluated the translational error in this experiment. As in previous cases, we conducted a series of ablation experiments and the results are shown in Table 3.

Our method achieved an accuracy within the range of 5 to 8 centimeters in all tested cases. Using the optimization term $E_{cc}$ consistently reduced the error in both datasets, while the optimization term $E_{cm}$ rarely improved the result. The use of control points slightly improved the accuracy. Figure 5 shows the control points employed. As a conclusion of this test, we can indicate that the joint optimization of all terms resulted in improvements for both datasets over the use of reprojection errors alone.

### 4.4. Corridor

**Figure 6:**
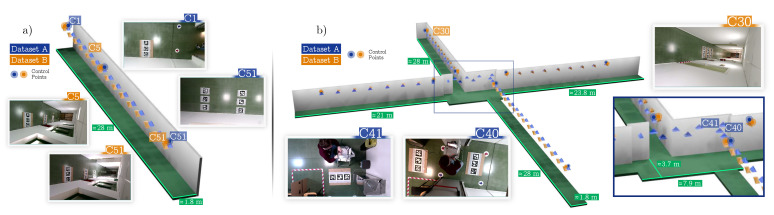
**Corridor and complete floor datasets**. Representations of camera positions and orientations are depicted using blue and orange pyramids. (**a**) Corridor datasets. (**b**) Complete floor datasets.

In this experiment, we tested our method in a real 50 m^2^ corridor (see Figure 6a). The setup involved a set of 15 cameras (of the same model as in the previous experiment) arranged linearly along the ceiling so they had minimal overlap with their immediate neighbor. Then, every camera group was formed of every pair of cameras with overlapping views. This configuration tested the precision of our method in maintaining spatial continuity across minimal overlapping fields of view. The ground truth was established again through manual annotation.

**Figure 7:**
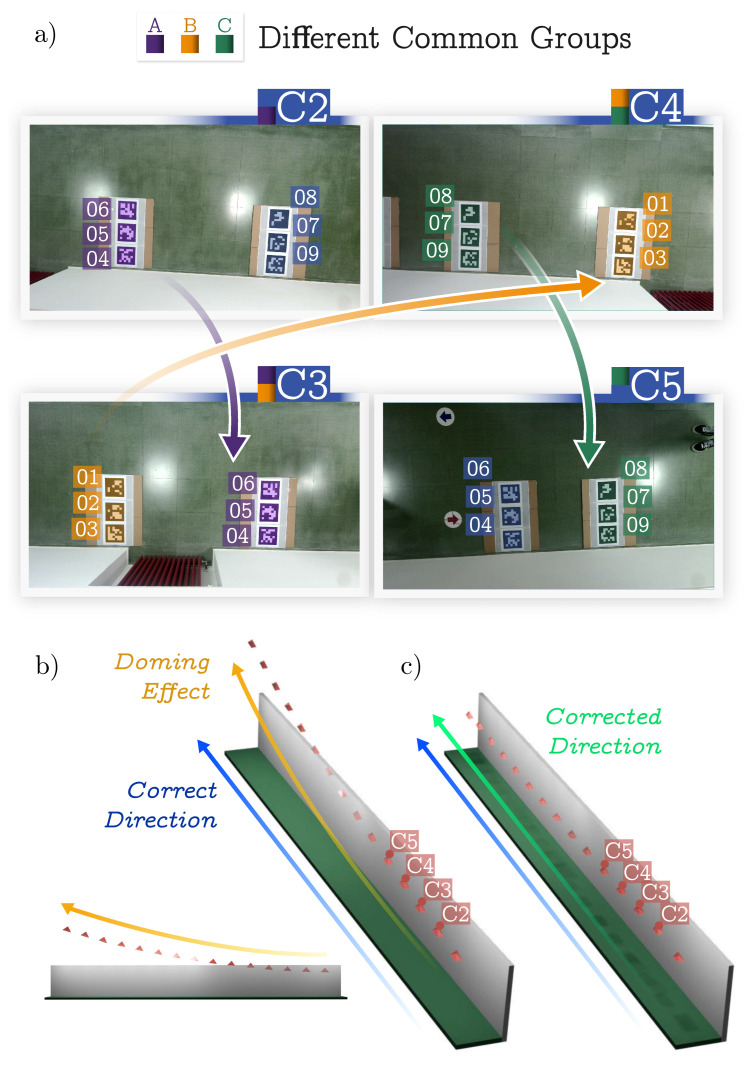
Reconstruction processes of corridor Dataset A, illustrating the impact of the control points and plane assumptions on the accuracy. Cameras are depicted as red pyramids. (**a**) Images of the dataset showing how markers were reused to create it. See the text for further details. (**b**) Baseline reconstruction without optimization, showing the initial deformations. (**c**) Enhanced reconstruction using the optimization terms $E_{cp}$, $E_{cc}$, and $E_{cm}$, which incorporate the control points and the assumption that cameras and markers were coplanar, significantly reducing the doming effect deformation and improving alignment.

We used only nine different markers (21.7 cm) to record the datasets using the following approach visually explained in Figure 7a. The markers were placed to be visible to two adjacent cameras (C2 and C3), and we captured an image with both of them. Then, we moved some of the markers (the closest to C2) to be visible to the next camera (C4). The process was repeated until the whole dataset was recorded using a minimal number of markers. If unique markers had been used (as required by MarkerMapper), a total of 45 markers would have been necessary for this dataset. For this reason, MarkerMapper was not tested in this experiment.

Again, we created two datasets, A and B. In Dataset B, the cameras were tilted at an oblique angle to the floor, enhancing the coverage, whereas in Dataset A, the cameras faced directly downward. Refer to Figure 6a for a visual representation of these configurations.

The results obtained are shown in Table 4. As in the previous case, we performed an ablation study that analyzed the impacts of the different optimization terms on the results. As can be observed, the errors obtained in Dataset A were high. The reason for this was the minimal overlap between the cameras, whose relationship could only be obtained by three markers that were very close to each other. On the other hand, because of the angles of the cameras in Dataset B, every pair of cameras saw at least six common markers.

Additionally, the optimization approach for Dataset A was conducted pair-wise, meaning each camera only shared markers with one adjacent neighbor. In contrast, Dataset B employed a triadic connection strategy due to the camera angles, allowing each camera to share markers with two neighboring cameras.

From the three-dimensional reconstruction of Dataset A using the baseline (see Figure 7b), one can observe a deformation in the camera’s estimated position, which describes a parabola. This is the well-known *doming effect* [33].

The deformation accumulated along the system, leading to a visible curvilinear distortion in the model. The configuration was adjusted by assuming that the cameras and markers were coplanar to address these potential deformations in such environments. Additionally, control points were strategically placed at both the beginning and end of the corridor, as shown in Figure 6. These control points provide a method with verifiable real-world data points essential for refining the reconstruction process.

With these adjustments, the revised method achieved satisfactory reconstruction results comparable with those in other tested environments. This highlights the importance of incorporating environmental specifics and control measures in complex setups to enhance the reconstruction algorithm’s accuracy and reliability.

For Dataset B, where this deformation was not observed, applying different optimization flags allowed for further solution improvement. This demonstrated that our method, particularly with the added adjustments for planarity and control points, was robust across different challenging scenarios, thereby validating its effectiveness in spatial reconstruction under constrained conditions.

### 4.5. Complete Floor

This experiment introduced a more complex environment that consisted of four long corridors that converged at a central room, covering approximately 214 m^2^ (see Figure 6b). A total of 51 cameras of the same type as in the previous experiment were placed on the ceiling, and their position was manually annotated. As in previous cases, two datasets were created. In Dataset A, cameras pointed straight down to the ground, while in Dataset B, they had an inclination of 45°. As in the previous experiment, we used only nine 21.7 cm markers that were moved to connect the cameras. To use MarkerMapper, a minimum of 169 unique markers would have been required.

The results of the ablation study of our method are shown in Table 5. The baseline results show a pattern similar to that observed in the corridor experiment, with a noticeable doming effect that affected the reconstruction accuracy. This deformation was primarily due to the initial camera arrangement and the complex geometry of the environment. As demonstrated in previous sections, applying optimizations, such as using control points (shown in Figure 6) and the assumption of coplanarity in cameras and markers, significantly enhanced the reconstruction quality.

With these strategic adjustments, our method successfully reduced the translational error to approximately 15 cm in this challenging environment marked by its intricate layout and large area. This improvement is particularly significant, considering the initial high errors of 134.45 cm and 248.35 cm in Datasets A and B, as outlined in Table 5. The refined results validated the robustness of our method, demonstrating its capability to adapt and perform effectively across diverse and complex scenarios.

### 4.6. Comparison with Other Works

Up to this point, we have evaluated our method under different challenging scenarios. Now, we compare it with some state-of-the-art methods. In particular, we compared it with the structure from motion (SFM) implementations OpenDroneMap [26], Pix4D [25], and COLMAP [27]. We also compared it with the Gaussian splatting approach PolyCam [31], and with the fiducial-marker-based approach MarkerMapper [17].

Despite numerous attempts, we were unable to make the methods OpenDroneMap, Pix4D, COLMAP, and PolyCam produce valid reconstructions. Although some methods produced partial reconstructions, they could not obtain a full reconstruction for any dataset. We believe the reason was the insufficient overlap between the cameras and the lack of distinctive keypoints to establish reliable matches. The only method that was able to work in the dataset was MarkerMapper.

Table 6 presents the results obtained, where the symbol ‘−’ indicates that a particular error was unavailable because we did not have the ground truth, and the symbol ‘×’ indicates that the method could not be applied. This was the case with MarkerMapper, which could not be applied in the corridor and complete floor datasets since we reused markers. The last column represents the results obtained by our method, which are presented in previous sections.

For the synthetic corridor, the reconstruction MarkerMapper obtained using Dataset A was similar to ours. Still, it was worse than our solution using Dataset B. MarkerMapper generally obtained slightly lower rotational errors than our method. While our method consistently obtained solutions comparable or superior to those generated by MarkerMapper, this advantage became particularly significant as the complexity increased, notably in terms of the translation error. In the first room, our method yielded consistent solutions for both camera arrangements. In contrast, for the first-floor and two-floor datasets, our method achieved better results than MarkerMapper, except for slightly worse rotational errors on the last ones.

In the real dataset for adjacent rooms, our method outperformed MarkerMapper. In the corridor and complete floor datasets, our method was the only one capable of computing the camera poses accurately. However, it is important to note that as the complexity of the task increased, the error margin in our method also grew.

Our experiments show that computing the poses of cameras with little overlap remained challenging, and few methods excelled under difficult conditions. Our analysis shows that our method could generate good results, while most alternatives often failed, particularly in environments with poor camera overlap or large distances. It proved particularly effective in complex scenarios, delivering superior accuracy and robustness.

## 5. Conclusions

This paper proposes a novel method for estimating the pose of a large set of fixed cameras in indoor environments. Our method enhanced the robustness of marker-based systems while preserving their inherent advantages, such as the capability to use a small subset of printed markers across various settings and the addition of real-world information to our solution. Our results demonstrate that our approach not only met but often exceeded the performance of existing state-of-the-art methods, particularly in challenging scenarios characterized by limited camera overlap and sparse environmental features. Moreover, our method effectively resolved the *doming effect* by incorporating real-world information, thereby improving accuracy and robustness in camera pose estimation.

Continued development of our camera pose estimation approach will focus on several key enhancements. We aim to expand the use of various types of markers, including the fiducial objects [19], to increase the flexibility of our system. This expansion will allow for improved adaptability across diverse camera arrangements and environments, enabling the possibility of having no overlap between cameras. In addition, we plan to incorporate advanced machine learning algorithms to tailor the bundle adjustment process for each specific case effectively. These advancements are anticipated to refine the accuracy and efficiency; reduce human intervention; and accelerate decision-making in real-time applications, such as augmented reality and autonomous indoor positioning.

## Figures and Tables

**Table 1:** **Results using the synthetic corridor datasets**. Lower values for both $E_{t}$ and $E_{r}$ represent a better reconstruction. The term $E_{rp}$ refers to the baseline, the term $E_{cp}$ refers to the usage of control points, $E_{cc}$ refers to the enforcement of cameras being at the same plane, and $E_{cm}$ applies the same principle to the markers.

	Synthetic Corridor			
	Dataset A		Dataset B	
Method	$E_{t}$ (cm)	$E_{r}$ (°)	$E_{t}$ (cm)	$E_{r}\;{(}^{\circ })$
$E_{rp}$ + $E_{cc}$	0.63	0.030	0.46	0.022
$E_{rp}$ + $E_{cm}$	3.12	0.118	6.08	0.095
$E_{rp}$ + $E_{cc}$ + $E_{cm}$	0.61	0.118	0.46	0.029
$E_{rp}$ + $E_{cp}$	1.67	0.116	2.65	0.165
$E_{rp}$ + $E_{cp}$ + $E_{cc}$	0.64	0.056	0.39	0.090
$E_{rp}$ + $E_{cp}$ + $E_{cm}$	1.69	0.056	2.63	0.166
$E_{rp}$ + $E_{cp}$ + $E_{cc}$ + $E_{cm}$	0.66	0.052	0.41	0.063

**Table 2:** **Results using the synthetic duplex datasets**. Lower values for both $E_{t}$ and $E_{r}$ represent a better reconstruction. Each synthetic duplex dataset was divided into three complexity levels: a single room, the entire first floor, and the complete duplex. The term $E_{rp}$ refers to the baseline, the term $E_{cp}$ refers to the usage of control points, $E_{cc}$ refers to the enforcement of cameras being on the same plane, and $E_{cm}$ applies the same principle to the markers.

	1st Room				1st Floor				2 Floors (Complete)			
	Dataset A		Dataset B		Dataset A		Dataset B		Dataset A		Dataset B	
Method	$E_{t}$ (cm)	$E_{r}$ (°)	$E_{t}$ (cm)	$E_{r}$ (°)	$E_{t}$ (cm)	$E_{r}$ (°)	$E_{t}$ (cm)	$E_{r}$ (°)	$E_{t}$ (cm)	$E_{r}$ (°)	$E_{t}$ (cm)	$E_{r}$ (°)
$E_{rp}$	0.14	0.022	0.50	0.013	1.08	0.159	6.61	0.367	6.56	0.235	9.77	0.379
$E_{rp}$ + $E_{cc}$	0.15	0.025	0.30	0.015	0.71	0.164	5.85	0.325	6.94	0.227	18.01	0.393
$E_{rp}$ + $E_{cm}$	0.14	0.016	0.24	0.040	1.07	0.160	8.98	0.395	3.73	0.113	14.99	0.575
$E_{rp}$ + $E_{cc}$ + $E_{cm}$	0.15	0.023	0.24	0.039	0.71	0.165	5.80	0.227	3.71	0.113	11.44	0.493
$E_{rp}$ + $E_{cp}$	0.13	0.024	0.22	0.020	0.79	0.152	7.09	0.506	4.81	0.334	12.58	0.390
$E_{rp}$ + $E_{cp}$ + $E_{cc}$	0.21	0.021	0.20	0.014	0.46	0.163	2.65	0.268	3.31	0.272	11.62	0.369
$E_{rp}$ + $E_{cp}$ + $E_{cm}$	0.13	0.020	0.21	0.053	0.76	0.159	6.62	0.444	2.73	0.044	10.71	0.535
$E_{rp}$ + $E_{cp}$ + $E_{cc}$ + $E_{cm}$	0.19	0.021	0.18	0.053	0.49	0.157	2.69	0.241	2.04	0.050	7.29	0.493

**Table 3:** **Results in the adjacent rooms datasets**. Lower values for both $E_{t}$ and $E_{r}$ represent a better reconstruction. The term $E_{rp}$ refers to the baseline, the term $E_{cp}$ refers to the usage of control points, $E_{cc}$ refers to the enforcement of cameras being at the same plane, and $E_{cm}$ applies the same principle to the markers.

	Adjacent Rooms	
	Dataset A	Dataset B
Method	$E_{t}$ (cm)	$E_{t}$ (cm)
$E_{rp}$	7.32	8.07
$E_{rp}$ + $E_{cc}$	6.63	4.99
$E_{rp}$ + $E_{cm}$	6.67	8.41
$E_{rp}$ + $E_{cc}$ + $E_{cm}$	6.58	5.10
$E_{rp}$ + $E_{cp}$	6.61	8.01
$E_{rp}$ + $E_{cp}$ + $E_{cc}$	6.60	5.60
$E_{rp}$ + $E_{cp}$ + $E_{cm}$	6.63	7.98
$E_{rp}$ + $E_{cp}$ + $E_{cc}$ + $E_{cm}$	6.63	5.62

**Table 4:** **Results using the corridor datasets**. Lower values for both $E_{t}$ and $E_{r}$ represent a better reconstruction. The term $E_{rp}$ refers to the baseline, the term $E_{cp}$ refers to the usage of control points, $E_{cc}$ refers to the enforcement of cameras being on the same plane, and $E_{cm}$ applies the same principle to the markers.

	Corridor	
	Dataset A	Dataset B
Method	$E_{t}$ (cm)	$E_{t}$ (cm)
$E_{rp}$	26.19	6.95
$E_{rp}$ + $E_{cc}$	1.22	1.07
$E_{rp}$ + $E_{cm}$	16.65	7.31
$E_{rp}$ + $E_{cc}$ + $E_{cm}$	1.60	1.01
$E_{rp}$ + $E_{cp}$	10.62	4.55
$E_{rp}$ + $E_{cp}$ + $E_{cc}$	1.84	0.93
$E_{rp}$ + $E_{cp}$ + $E_{cm}$	3.95	4.14
$E_{rp}$ + $E_{cp}$ + $E_{cc}$ + $E_{cm}$	1.78	0.84

**Table 5:** **Results using the complete floor datasets**. Lower values for both $E_{t}$ and $E_{r}$ represent a better reconstruction. The term $E_{cp}$ refers to the usage of control points, $E_{cc}$ refers to the enforcement of cameras being on the same plane, and $E_{cm}$ applies the same principle to the markers.

	Complete Floor	
	Dataset A	Dataset B
Method	$E_{t}$ (cm)	$E_{t}$ (cm)
$E_{rp}$	133.97	247.80
$E_{rp}$ + $E_{cc}$	79.22	167.11
$E_{rp}$ + $E_{cm}$	123.72	238.88
$E_{rp}$ + $E_{cc}$ + $E_{cm}$	78.05	167.67
$E_{rp}$ + $E_{cp}$	36.13	42.00
$E_{rp}$ + $E_{cp}$ + $E_{cc}$	14.76	16.40
$E_{rp}$ + $E_{cp}$ + $E_{cm}$	36.75	22.80
$E_{rp}$ + $E_{cp}$ + $E_{cc}$ + $E_{cm}$	14.82	15.72

**Table 6:** **Comparison with other works**. Lower values for both $E_{t}$ and $E_{r}$ represent a better reconstruction. MarkerMapper was the only method capable of obtaining coherent results. The symbol ‘−’ indicates that a particular error was unavailable because we did not have the ground truth, and the symbol ‘×’ indicates that the method could not be applied.

		Methods MarkerMapper [17]	Our Method		
Datasets		$E_{t}$ (cm)	$E_{r}$ (°)	$E_{t}$ (cm)	$E_{r}$ (°)
Syn. corridor	A	0.36	0.004	0.66	0.052
	B	36.37	0.018	0.41	0.063
Syn. duplex 1st room	A	0.13	0.003	0.19	0.021
	B	0.54	0.004	0.18	0.053
Syn. duplex 1st floor	A	1.19	0.003	0.49	0.157
	B	4.09	0.022	2.69	0.241
Syn. duplex 2 floors	A	18.19	0.090	2.04	0.050
	B	14.94	0.670	7.35	0.552
Adjacent rooms	A	6.77	−	6.63	−
	B	34.55	−	5.62	−
Corridor	A	×	−	1.22	−
	B	×	−	0.84	−
Complete floor	A	×	−	14.82	−
	B	×	−	15.72	−

## Data Availability

Publicly archived datasets and code can be found at https://www.uco.es/investiga/grupos/ava/portfolio/indoor-camera-positioning/ (accessed on 29 June 2024).
